# Epinephrine-Containing Topical Anesthetic Gel Inducing Systemic Epinephrine Toxicity: A Case Report

**DOI:** 10.7759/cureus.44844

**Published:** 2023-09-07

**Authors:** MD J Fateha, Philip Willsie

**Affiliations:** 1 Emergency Medicine, Jefferson Health New Jersey, Stratford, USA; 2 Critical Care, Jefferson Health New Jersey, Stratford, USA

**Keywords:** non-st segment elevation myocardial infarction (nstemi), lactic acidosis, systemic epinephrine toxicity, numbing gel, tattoo, tntk, topical anesthetic gel, epinephrine toxicity, epinephrine

## Abstract

Systemic epinephrine toxicity is a rare complication following inadvertent, excessively large, rapid subcutaneous, intramuscular, or intravenous administration. Signs and symptoms of epinephrine toxicity include the rapid onset of transient agitation, hypertension, tachycardia, lactic acidosis, and dysrhythmias with potentially fatal consequences.

This is a case report of a 33-year-old female who experienced epinephrine toxicity following the use of a topical anesthetic cream containing lidocaine and epinephrine. The patient had multiple applications to her chest before and during tattoo placement, which led to tachycardia, elevated blood pressure, headaches, chest pain, nausea, vomiting, and anxiety. The patient was brought into the ED, where her vital signs had begun to normalize, but laboratory analysis was concerning for severe lactic acidosis and non-ST elevation myocardial infarction. After admission to the hospital, the patient’s symptoms quickly improved, the lactic acidosis resolved, and further workup was unrevealing. This case report explains the potential adverse outcome of topical epinephrine use to help create awareness of the possibility of systemic epinephrine toxicity.

## Introduction

Epinephrine is an adrenergic agonist used to treat hypotension, bradycardia, cardiac arrest, anaphylaxis, and bronchospasm [1,2]. Epinephrine is most typically administered through intravenous, intramuscular, and transdermal routes. Epinephrine is also known to be absorbed via the trans-dermal route, but the absorption is typically limited, and we can find no prior reports of systemic toxicity following the topical administration of epinephrine-containing solutions. Adverse reactions to epinephrine administration most commonly occur due to excessive dosing because of iatrogenic error [2]. Epinephrine overdose can lead to the rapid onset of agitation, hypertension, tachycardia, and dysrhythmias [2]. Symptoms of toxicity typically resolve rapidly given epinephrine’s short half-life; however, if symptoms are not improving, outcomes can be profound and fatal.

This case report was previously presented as a poster presentation at the Rowan-Virtua School of Osteopathic Medicine's Research Day on May 4, 2022.

## Case presentation

We present a case of a 33-year-old female with no medical history who was brought into the ED by emergency medical services (EMS) for chest pain. Three hours prior to arrival at the ED, the patient was obtaining a large tattoo over her upper anterior chest while at a tattoo convention. The EMS were called, and they found her vital signs to be as follows: sinus tachycardia at 130 beats per minute (bpm), blood pressure (BP) of 180/110 mmHg, oxygen saturation (Sp02) of 100% on room air, and respiratory rate (RR) of 26 breaths per minute.

Upon arrival at the ED 20 minutes later, her repeat vital signs were as follows: heart rate (HR) of 100 bpm, BP of 104/55 mmHg, Sp02 of 100% on room air, and RR of 20 breaths per minute. The patient was still experiencing chest pain, but her other symptoms were resolved. The patient's physical exam findings showed she was not agitated, her pupils were not constricted or dilated, no jugular venous distension was noted, and there was a freshly drawn tattoo on her chest but no signs of cellulitis or crepitus. Pertinent positive physical exam findings were significant only for mild tachycardia and tachypnea. The electrocardiogram in the ED showed normal sinus rhythm at 83 beats per minute, a normal axis, QRS: 96 milliseconds, QTC: 446 milliseconds, and no ST-segment or T-wave abnormalities. Laboratory analysis was significant for hypokalemia with a potassium level of 2.7 mmol/L. Acute kidney injury was seen with a creatinine of 1.24 mg/dL (prior creatinine level: 0.72 mg/dL), non-ST elevation myocardial infarction with a high-sensitivity troponin of 1272 ng/L increasing to 2777 ng/L three hours later, and elevated lactate at 7.9 mmol/L decreasing to 2.3 mmol/L three hours later. While in the ED, the patient was given 60 mEq potassium chloride, 2 L normal saline, and 81 mg aspirin. She was admitted to the intensive care unit (ICU) for close monitoring given her unexplained lactic acidosis to rule out pulmonary embolism, cardiogenic shock, and prinzmetal angina.

After admission, the patient described applying four tubes (40g) of "TKTX®", a topical anesthetic gel containing lidocaine and epinephrine, to her chest over the course of several hours. The initial tube was applied prior to tattooing, and shortly after, the patient stated she felt her heart racing. After the tattooing process started, additional tubes were applied over the course of the next couple of hours. Throughout the process, she felt her heart rate was elevated out of proportion to any discomfort or anxiety from the tattooing process. The rapid HR was also noted on visual inspection of her neck by the tattoo artist. After the application of the last tube, there was a break in tattooing to allow the gel to absorb, during which she began to experience severe anxiety, a generalized headache, chest pain, nausea, and vomiting.

While in the ICU, repeat laboratory analysis showed troponin peaked at 3946 ng/L. There was a resolution of her hypokalemia with a potassium level of 4.9 mmol/L and a resolution of acute kidney injury with a creatinine level of 0.72 mg/dL. The patient denied any illicit or over-the-counter drug use, and the urine drug screen was negative for cocaine, methamphetamine, phencyclidine, and tetrahydrocannabinol. Transthoracic echocardiography was performed and showed low-normal left ventricular systolic and diastolic function with no regional wall abnormalities to suggest myocardial infarction. On reassessment, the patient was resting comfortably and in no acute distress. She stated that her chest pain, headache, nausea, and vomiting had fully resolved. The patient was safely discharged home after 14 hours of observation.

## Discussion

We report the first documented case of epinephrine toxicity secondary to the use of topical epinephrine-containing anesthetic gel. Epinephrine is an adrenergic agonist known to induce vasoconstriction through increasing vascular smooth muscle contraction [3]. Epinephrine is absorbed into the systemic vasculature within one to two hours of topical application and can lead to vasoconstriction of the nearby vasculature [3]. Our patient experienced symptoms of toxicity within minutes of application that peaked after a few hours of use. Although we cannot be sure, the rate of transdermal absorption may have been hastened by the tattooing process itself and the effects enhanced by the proximity of the application to the patient’s heart.

When administered systemically, epinephrine is well known to increase heart rate and the force of myocardial contraction, resulting in tachycardia and elevated blood pressure, lower serum potassium levels, and increased lactate production. Our patient presented with tachycardia, hypertension, hypokalemia, and an elevated lactate level. Epinephrine has a half-life of only 11 minutes once in the bloodstream, so its effects after intravenous administration are fleeting [4]. Our patient’s symptoms persisted for more than 30 minutes after the last application. This prolonged duration is likely due to consistent transdermal absorption, leading to persistently toxic serum levels that diminished as the topical epinephrine concentration decreased and the systemic epinephrine was quickly metabolized upon reaching the bloodstream.

The gel used by the patient, TKTX®, is a widely available topical anesthetic cream that contains various concentrations marketed for topical anesthesia prior to tattooing or piercing. Our patient used a total of four tubes that each contained 10g of gel containing 7% lidocaine and 2% epinephrine. Significant systemic absorption of topical anesthetics resulting in toxicity secondary to lidocaine is well documented but results in hypotension, coma, seizure, and bradycardia [5]. None of our patient’s symptoms or findings were consistent with lidocaine intoxication, making it very unlikely to have been the culprit [6].

We performed a thorough review of existing medical literature as of July 2023 and found no documentation of epinephrine toxicity resulting from transcutaneous absorption prior to our case. Toxicity may have occurred in our patient and not others because of the use of four times the manufacturer-recommended amount. She also had tattooing performed on top of the skin where the gel was applied and then had the gel re-applied to a large area of freshly tattooed skin. This may have increased the speed and amount of systemic absorption due to the needling process disrupting the cutaneous barrier and, as a result, increasing perfusion to the area. It is also feasible that the proximity of the application to her heart could have facilitated some absorption via direct extension. This case helps raise awareness among medical providers about the potential for systemic epinephrine toxicity resulting after cutaneous application and helps to differentiate this from local anesthetic-induced systemic toxicity. Clinicians should be mindful that patients presenting with signs and symptoms signifying a hyperadrenergic state in the setting of topical anesthetic-based gel application could be suffering from systemic epinephrine toxicity. Similarly, the greater tattoo community should be made aware of the potential for this uncommon but potentially serious complication.

## Conclusions

This case report describes the first-ever documented occurrence of epinephrine toxicity following topical epinephrine-containing anesthetic gel use. In patients where clinicians suspect accidental epinephrine toxicity, we recommend obtaining an electrocardiogram and troponin to rule out dysrhythmia and myocardial infarction, a basic metabolic panel to investigate for significant hypokalemia, acidosis, or kidney injury, and a serum lactate level to help confirm the diagnosis. Consideration should be given to hospital admission with telemetry monitoring while further confirming the absence of other causes or organ damage.
